# On the Nature of Volition

**Published:** 1863-01

**Authors:** J. Lockhart Clarke


### MEDICAL CRITIC

AND

### PSYCHOLOGICAL

### JOURNAL.

JANUARY, 1863.

### Art. I.—ON THE NATURE OF VOLITION, PSYCHOLOGICALLY AND PHYSIOLOGICALLY CONSIDERED.

By J. Lockhart Clarke, F.R.S.

### Part II.

(*Continued from* No. viii. p. 592.)

After showing that *conscious intelligence* is the distinctive element of volition; that *pure sensation*, in man and the higher animals, is a state of consciousness entirely distinct and different in its nature from that of *intelligence*, and therefore cannot, *by itself*, constitute *volition;* I concluded my former article by explaining the manner in which, by *voluntary effort*, we learn to initiate, control, and co-ordinate our muscular movements. I shall now proceed analytically to inquire more fully into the *psychological character* of volition.*

It has ever been the opinion of some philosophers that *volition* or *voluntary effort* is the function of a *special faculty* termed the "Will," which is not only distinct and different in its nature from all other mental faculties, but also independent of them, by virtue of a peculiar and inherent power of *self-determination*. On the other hand, it is contended by a comparatively increasing number of philosophers, that no such *special faculty* exists for the performance of volition, any more than there exists a special faculty for walking, dancing, or any other particular kind of voluntary action; and that consequently the term "Will" is simply the general expression of our ability to perform such actions by a special application and use of some of the *ordinary*

* See Note A, p. 15.

faculties of our minds. In this sense the term "Will" bears the same kind of relation to *voluntary* actions, as the abstract term "virtue" bears to *virtuous* actions.

Now the only way to form an accurate judgment on these two opposite opinions, and arrive at a correct conclusion on the psychological nature of volition, is to make a careful and complete analysis of the different kinds of voluntary actions, and in each kind, trace the separate parts of the process to their respective principles or source. We have already seen that reflex co-ordinate movements which have an evident design to fulfil, may be directly excited by *common sensation alone,* as their active or *dynamic* principle. Such are the reflex movements of winking, excited by the painful stimulus of light or some other kind of irritant; those of sneezing, from irritation of the lining membrane of the nose; and those of coughing, from irritation of the larynx or lungs. Now all these movements are specially designed for the exclusion or ejection of the offending irritant; but since the design which they involve does not *originate* from the *conscious intelligence* of the being in whom they occur, they cannot be accounted voluntary. But common *physical* sensation, within certain limits of intensity, may nevertheless become the direct or *immediate* dynamic agent in a true act of *volition.* This proposition which, at first sight, will appear paradoxical, may, however, be verified on further consideration. Suppose, for example, that I experience an acute pain, suddenly inflicted by a sharp instrument on my arm or leg. The instinctive and *immediate effect* of the pain will be *on the spinal cord,* and thence *on the muscles,* which retract the limb from the source of injury, and the entire process will be called a *reflex action arising from a painful impression.* Suppose, moreover, that the *dynamic tendency* of the pain to produce this reflex movement be *within the power of my control;* but that my understanding suggests no reason or motive *why it should be prevented,* that is, suggests no other *opposing* principle of action, or *dynamic feeling* of *greater influence* than the pain—then the retraction of the limb may be considered *voluntary;* for although it be merely a reflex action *through physical sensation alone,* it is nevertheless *approved of* and *permitted* by the understanding, or, in other words, is *not opposed* by any other feeling suggested by thought, and is therefore *equivalent* to the result of an express *wish* or *desire.* But the case has this peculiarity, that the movement *requires* no *effort* or *express desire* to perform it, for the necessary muscular co-ordinations, are, as may be seen in the new-born infant, already associated, from birth, with the corresponding and pre-arranged organic condition of the nerve centres; and therefore there is no difficulty or obstacle to be overcome by that *co-operation* and *concentration* of the *regulative* element of *intellect* with the *dynamic* element of *feeling*, which constitute our consciousness of *personal effort.* When, however, the movement, and therefore the necessary muscular co-ordinations required for the relief of the pain, differ from those which are *naturally* associated with sensation in simple reflex action, then the sensation must be reflected *through the intellect* or *regulative* faculty, and in *combination* with it will be felt as a *special desire,* attended with a sense of *effort,* to accomplish the end in view.

Let us again suppose that I experience an *appetite* which it is in my power to gratify at once. The appetite, requiring for the attainment of its object the co-operation of the senses and intellect, will be reflected through the latter in the form of *special desire,* which, *in the absence of any other opposing feeling* suggested by thought, *will necessarily and of course,* under the direction of intelligence, exert its influence on my bodily organs, and effect, with a sense of *effort,* those muscular co-ordinations which are required for the end proposed. The movements, therefore, must be *voluntary,* since they are performed with a conscious design, under the guidance of intelligence, which enables me to consider the circumstances of the case, and to judge whether or not the action be consistent with stronger feelings of my nature, as, for instance, with my regard for self-preservation, well-being, propriety, &c.

We see then, that in these two simple examples of voluntary action, there is no *special faculty* at all corresponding to the term "Will;" but that the process consists of the combined operation of two of the ordinary faculties of our nature, viz., 1. Of either *common sensation* or of *appetite,* which I shall designate *dynamic* or *conative* elements of volition; and 2. Of *conscious intelligence,* which I shall call the *regulative* element of volition.

It may be inferred, then, that in both these kinds of *voluntary* actions the process is often as completely reflex as in those *involuntary* actions which result from *sensation alone.* In the latter, when a painful impression, for example, is made on a given part of the foot or leg, the sensational centre, on receiving that impression, will react on the muscles which retract the limb, but *only* in this way. The sensational centre, however, when excited by the *same* impression, may react in the *same* direction on the *same* muscles, with the *approval* of the *understanding;* and then, as I have already shown, the action may be voluntary; but besides this it may react in an entirely different direction and on different muscles, through the intellect, with the intention of *removing the whole body* from the source of injury. Yet the association of the reaction with intelligence, and the mere *difference in the direction* which it may consequently take, do not render it at all the *less reflex.* And similarly in the case of appetite. The nervous centre in which it is experienced, on receiving through the senses the impression from its appropriate object, reacts, according to circumstances, under the guidance of intelligence and the organs of special sense, on the particular muscles that may be necessary to secure the object in view; and, however circuitous and complicated the resulting processes may be, or however long the time that they may occupy in accomplishing their end, the actions themselves are as much *reflex* as are those simple and *in*voluntary reactions which *immediately* follow upon physical sensation alone, or even without sensation. There is this difference, however, between *common sensation* and *appetite* with regard to reflex action—that while the former, in a normal state of the organism, can *always* be excited by its appropriate stimulus,—the *special susceptibility* of the latter is only *occasional,* because it depends on the variable condition of certain internal organs. Thus pain, in a normal state of the body, can always be produced by the application of a sharp instrument; while the appetite of hunger and its consequent reaction can be excited only when the system *indirectly* and the stomach *directly* demands a supply of food. Voluntary actions, however, of which *appetite* is the dynamic element, have more often a *centric* origin; as when we are impelled by a feeling of hunger, independently of the *presence of food;* and the process is sometimes similar in the case of *common sensation;* as when we are excited to action for the relief of pain which is not immediately dependent on external irritation. But still, *always* in the case of appetite, and *mostly* in the case of common sensation, the action must be accounted *reflex;* for the appetite, as experienced in its nervous centre, is always occasioned by the condition of some other organ; and the pain experienced in the *sensorium* is most frequently excited by irritation of some other part of the body.

If the impulse or tendency of the appetite to produce *action* be unopposed by any *outward* obstacle, or by any other *internal* impulse excited by reflexion, it will at once spend its force, under the guidance of intelligence, in effecting the movements necessary to obtain its object or end. But if it be checked in its active tendency by any *outward* obstacle, which it is possible and worth the trouble to overcome, it then manifests itself in the sensorium, as an uneasy sensation of craving, which now operates, first, internally, by stimulating the intellectual faculties to devise the means of gratification. This craving, or urgent feeling of attraction towards a definite object or end, in a being endowed with independent and conscious intelligence, constitutes what is called *desire.* But an *action* or movement *produced* by this craving or impulse of appetite, when *guided by intelligence,* is an act of *volition.* A voluntary action, therefore, in this case, is nothing more than the direct and *dynamic effect of a desire to perform it,* with the intention or conscious end of gratifying the appetite by securing its object. A craving or impulse towards a definite end occurs in the case of *pure instinct;* but since it is not accompanied by an independent intelligence, it cannot be contemplated in relation to the *future* or the *past,* or viewed by the light of experience. The end, therefore, is not a *conscious* end; and the impulse is a blind and fatal impulse, which must necessarily result in the *particular action to which it tends,* unless it be opposed by some external object or impression; so that there can be no selection, no choice or control, and therefore no true volition.* But, in man, the *tendency* of a desire, arising from appetite, to result in *action* that may be voluntary, although not immediately opposed by *external* force, may nevertheless be *restrained* by some other *internal* impulse, or dynamic feeling excited by memory, or by reasoning, from the associations of experience, on the circumstances of the case. Thus, on recalling a painful sensation or unfortunate event, as the result of a former indulgence of the same appetite under similar circumstances, the *fear* of *similar consequences, from a natural aversion to pain,* may, if sufficiently strong, not only *oppose* and *check* the *volitional tendency* of the original *desire to act,* but may *impel* to the performance of other actions, of which the object is *to avoid a threatened punishment:* as when an individual about to commit an act of theft, *from the impulse of hunger,*—on finding himself detected, is not only *restrained* by *fear,* but is *impelled* by the *same feeling* to *make his escape* from the probable, or perhaps, the impending consequences of his detection. I have said "the *volitional* tendency of the original desire to act;" for although that tendency or impulse arises from simple *appetite* as a dynamic element, yet under ordinary circumstances and the guidance of conscious intelligence, the action which it would necessarily excite, unless *restrained* by some *other dynamic feeling* suggested by that intelligence, would have all the characters of a true and perfect *volition,* and would be treated as such by the law. Here, then, we have another instance of *volition,* of which the *dynamic* or *active* element is *fear,* excited by thinking of the evil conse-

* See Note B, p. 16.

quences that might result from a *voluntary* action performed from the impulse of appetite, with a view to its gratification. The love of something good, with a craving, or feeling of attraction towards it, and a consequent *desire to act,* for the purpose of obtaining it, is opposed by a dislike and dread of impending evil, with a feeling of repulsion, which, in conjunction with intelligence, is equivalent to a *desire of avoiding it.* If a pain, punishment, or other evil, be expected to ensue only on the *gratification* of a desire, the thought of the consequences of such indulgence may be only just sufficient to restrain *outward* action, while the longing or craving remains urgent; but if the mere *existence* or *feeling* of a desire—independently of its gratification—such as that excited by a sinful thought, be expected to entail future punishment, then the *feeling* itself, or the inclination, may be subdued by the *opposing force of fear;* or, in other words, the natural or instinctive impulse towards anticipated good, which, *if unrestrained,* would have been the *dynamic* element of a *voluntary act,* may be *either checked in its action,* or *superseded as a feeling,* by the natural dread of anticipated evil, which, in its turn, may now become the *dynamic* element of an *actual* volition; for although the feeling of dread may not excite any *outward movement,* it will nevertheless exert a certain force in restraining *that tendency* in the desire which it controls, and this *control* of one *feeling* by another, through reflection, no less than the movement which it may excite, is acknowledged, on all hands, to constitute an *act* of *volition.* A desire, however, of any kind, although checked in its *tendency* to action by the superior force of fear, may be *reinforced* by some other dynamic feeling suggested by further reflection, and which, by *opposing* the *restraining* influence of *fear,* may leave the *dynamic element* or *feeling* of the *original* desire free to excite the originally-*intended act* of volition. Let us suppose, for instance, a person, impelled by a feeling of humanity, proceeding to rescue another from some perilous position. Finding that the attempt would be attended with considerable danger to himself, he might at once be *deterred by fear;* but a sense and impulse of duty, suggested by reflection, might be strong enough to destroy the *opposing influence,* and, perhaps, the actual *feeling,* of *fear,* and thus help the *original* desire, arising from humanity, to go off into action. Or if a sense of duty, alone, were insufficient, some other feeling, such as shame, from the imputation of cowardice, or ambition, a prospect of reward or interest, might contribute to turn the scale in favour of humanity. Here, then, we have a complex form of volition, the resultant of a train of dynamic feelings of which each was excited in succession, and modified, replaced, or overborne by another, through the medium of reflection on their respective ideas. In like manner, the emotions of anger, envy, hatred, jealousy, love, benevolence, justice, &c., give birth respectively to *special desires of action*, and may oppose, modify, or reinforce each other, successively or alternately, in very complex or composite acts of volition.* The dynamic *tendency* of anger, for instance, may be restrained by a feeling of *interest;* by *fear* of the consequences of indulgence; by a feeling of *compassion* arising from reflection on the unfortunate condition of the offender; or by *gratitude* and *regard*, from recollection of kindness formerly received. The dynamic tendency of *jealousy* or *hatred* may, in like manner, be opposed and overborne by the volitional force of *pride, shame*, or *justice*, according to the moral peculiarities of the individual, and the manner in which the circumstances of the case are contemplated. When a father indulges in habits and inclinations which are inconsistent with the welfare of his family, and which his love for them alone is insufficient to restrain, a consideration of *duty* and a consequent sense of guilt, arising from reflection on his position and obligations, may suffice, in conjunction with the love of his family, as a volitional check to the urgency of his unnatural desire. In cases of this description there is often a struggle of variable severity and length between several antagonist and contending dynamic feelings, one subduing or diminishing the force of another, or gaining and losing alternately a momentary advantage, according as the ideas which excite them succeed each other in the mind; for the strength and volitional force of the opposing feelings will depend, partly on the comparative *vividness* of the ideas, and partly on the susceptibility of the individual for the feelings which they excite. While one whose susceptibility is sufficiently alive may be incapable of realizing, with sufficient distinctness, the ideas whieh excite it, and of keeping them steadily in view with sufficient vividness to produce the required intensity of feeling; another, with deficient susceptibility, may have a vivid conception and complete command of the correlative ideas, without experiencing the intensity of feeling which they would excite in the former case. Thus, an individual about to commit some unlawful act may fully realize in *idea*, or *know* perfectly, the amount of guilt which he is about to incur, but is unable to *feel* it in a corresponding degree, or in the degree in which it would be felt by ordinary men under similar circumstances. Sometimes in the same person there is great susceptibility of feeling with great command or vivid conception of the *correlative ideas;* and sometimes there is a

---

* See Note C, p. 17.

deficiency of both, as in the melancholy cases of many criminals and idiots.

We see then, that in volition, the *activity*, or *active part*, of the process, is the immediate effect of some kind of feeling, and of the *same* kind of feeling as that which excites *in*voluntary action; so that the only difference between the two kinds of *processes* is, that in *volition* the active feeling is accompanied and modified by the regulative element of intelligence, through which it may be either restrained by some *other* feeling, or guided to a conscious end. The feelings or *active* elements which are thus common to both voluntary and *in*voluntary acts, consist, as I have shown—1. of common or physical sensation acting either with the assent or with the guidance of the understanding; 2. of one of the bodily appetites acting under the direction of intelligence; 3. of one of the emotions, acting under the same conditions, or only by the *assent* of the understanding. What particular *kind* of these feelings, or what particular feeling of the kind, when brought into opposition with others, shall *prevail* as the active or dynamic element of a volition, will depend on the following conditions: 1. on the difference in the natural *excitability*, *intensity*, and *duration* of the several dynamic feelings, *in relation to each other;* 2. on habitual indulgence, or the relative frequency with which any of those feelings have been allowed to operate as the dynamic or active elements of *volition;* 3. on the relative amount of real or supposed good or evil presented to the senses or represented to the mind, as belonging to the objects by which these feelings are excited. The *understanding, alone*, or a process of *pure intellect*, has no direct power of exciting a true act of volition. A *feeling* of some kind, in combination with it, must be the motor or dynamic agent. The *idea* of an object, capable of affording pleasure, never excites a *voluntary action*, unless there be a corresponding *feeling* or *appetite*, from which there arises a *desire to act* in reference to that object. And the *idea* of pain will fail to excite a voluntary movement, unless there be some *fear* of its *affecting* us, and a consequent *desire to avoid it.* The most vivid *idea* of food would never of itself excite the movements necessary to obtain it, if the corresponding or appropriate *appetite* were wanting. It is true that a particular idea, or a series of ideas, by frequent association with muscular actions, may excite respectively a particular movement or series of movements without the mediation of any explicit *desire* or *intention;* but such movements are secondarily *automatic* or *involuntary*.* On the other hand, one emotion or desire has no *direct* power of

* See Note D, p. 18.

exciting another : it can do so only through the medium of the understanding, by which the objects fitted to excite the desired affection are presented to the senses, or represented as ideas. If I desired to excite in myself a feeling of *charity* or *pity*, I could succeed only by intentionally either visiting personally, or calling to mind, the objects of these affections. But the desire itself decreases just in proportion as it raises the new affection, and when satisfied, is replaced entirely by it. In like manner, whenever two affections are opposed through the medium of the understanding, the one increases at the expense of the other. Suppose, for example, that I feel an impulse or desire urging me to commit an action which is associated with the *idea,* and through this, with the *feeling* of shame; since I cannot attend to two distinct and different *thoughts,* and have two distinct and different *feelings,* at one and the same instant of time,—in proportion as my attention is directed to the ideas which excite the feeling of *shame,* it is withdrawn from the objects or ideas which excite the desire for the *shameful action;* or in other words, the *force* of my *desire to perform the shameful action* is *inversely* as the *feeling of shame,* suggested by reflexion on its correlative ideas; and conversely, the *feeling of shame* will *decrease* just in proportion as the understanding is busy with the ideas which excite *the desire to perform the shameful action.** The suspension, for a time, of an act of volition by this process of balancing one dynamic feeling against another, through the medium of reflexion, or the ideas which respectively excite them, constitutes an act of *deliberation,* or, as the term implies, a mental process of *weighing* the *relative amount* of good, of evil, or of good and evil, in two or more objects of undetermined value, in consequence of a natural tendency to select the *most eligible,* or *that* which either affords the *greatest* amount of *pleasure,* or inflicts the *least* amount of *pain.* When this has been fairly determined, the volitional tendency or desire, having no longer any reason for hesitation, will proceed at once to *choose*—that is, exclusively *fix upon* the object or end which the judgment of the understanding has *indicated* as the *most* eligible, and if expedient, will forthwith excite the movements necessary to obtain it—precisely in the same way as it would have done, if the *selected object or end* had *alone* been *originally* presented to the attention, and there had, therefore, been no *opportunity* of *choice,* and consequently no need, or rather no *possibility,* of *deliberation.* The immediate cause of deliberation, therefore, is a desire to obtain the greatest amount of good, or the fear of incurring evil by hasty

* See Note E, p. 22.

volition, when the circumstances of the case are imperfectly known. To this cause or state of mind we give the name of *caution,* which always results from experience, but is naturally a stronger feeling in some persons than in others. The simplest kind of *deliberation* is when the objects or circumstances to be weighed are cognizable by the senses; small in number; all of the same quality, that is, either *good* or *evil,* and different only in *amount* or *degree ;* and the process becomes more complicated, difficult, and prolonged, not only in proportion to the *number* and *variety* in *quality* and *degree,* of the circumstances of the case, but in proportion, also, to the *lapse of time* between any *past* and *present* experiences which it may be necessary to compare together; for the more *recent* the experience of a pleasure or a pain, and the more *vivid* its *idea,* the greater is its *weight* in deliberation. But however complicated and prolonged the process of *judgment* may be, it does not, as already stated, alter the true relation between the *object* or *circumstances* upon which it has decided, and the corresponding *desire* or *aversion* which is the active *feeling* or dynamic element in the volitional act. The understanding *alone* does not *excite* the action or movement, but only *guides* or directs it after pointing out the *existence* and *amount* of *good* or *evil* to be sought or avoided by the appetite or feeling.* These two elements, then, or principles of our nature, the *intellectual* and the *æsthetic* or *orectic,* which *together,* and *only* together, constitute "ourselves" and the consciousness of "ourselves" as personal agents, are all that can be discovered, and indeed, are all that can be *necessary* in any conceivable act of volition; so that all that can be, with propriety, understood by "the Will," is *the combination and co-operation of these constituent principles of our nature for a conscious and definite end.*

From the above analysis of different kinds of volition, or of voluntary actions, it follows, that what is commonly called a "motive" *to* "the Will," is nothing else than one of the *active feelings*—a *sensation,* an *appetite,* or an *emotion*—which, by reflection through the intellect, under favourable circumstances, constitutes the *dynamic element* of "the Will" itself, on any given occasion. Thus, when a person is said to *act* from a *sensual* "motive," the sensual *appetite* is the motor or active element which, by reflection through, and in conjunction with,

* Aristotle very properly remarked, that what *affirmation* and *negation* are in regard to the *reasoning* faculty, *pursuit* and *avoidance* are in regard to *appetite* or craving: Ἐστι δ' ὅπερ ἐν διανοίᾳ κατάφασις καὶ απόφασις, τουτ' ἐν ὀρέξει δίωξις καὶ φυγή. (*Ethic. Nicom.*, lib. vi. cap. ii.) He also very properly calls deliberate choice, a *craving guided by consultation* (ὄρεξις βουλευτικὴ), and either *orectic* (craving) *intellect,* or *craving accompanied with reason* (Διὸ ἢ ὀρεκτικὸς νοῦς ἢ προαίρεσις, ἢ ὄρεξις διανοητική.)—Ibid.

the *knowing* and *regulative* element, constitutes the "Will" on that particular occasion; and if the appetite, although experienced, should *not result in action*, it is only because it would be opposed and restrained by some other active feeling, suggested by reflection—as, for instance, the *feeling of shame*—which would then be the "motive" in the *volitional check*. In like manner, when a person is said to *act* from a *motive* of *interest*, from a *motive* of *compassion*, from a *motive* of *revenge*, or of *envy*, the real meaning is, that the *dynamic influence* of each of these feelings respectively, at the time of acting, is greater than that of any other feeling or feelings suggested by reflection, and is, therefore, the *active* or *motor* element of "the Will" for the time being. It is evident, then, that these "motives," or *motor feelings*, are not separate and distinct from "the Will"—which is usually supposed to be a separate source of action—but constitute, in some form or other, and within certain limits of intensity, the *dynamic elements* of *volition*. Whenever, therefore, we hear of *motives* being "addressed" *to* "the Will," the expression means nothing more than that they are addressed or submitted to the *judgment of the understanding*, and balanced in deliberation against any *other motives* or feelings that may be suggested by it, in reference to good or evil. And whenever it is said that a "motive" *influences* or *determines* "the Will," nothing more can be meant than that it *overcomes* any other feeling or motive that may be experienced at the moment of its presentation, and thus becomes, on that occasion, the *dynamic element* of "the Will." And when the process of deliberation is complicated or involved, the *power* of "the Will" *to act* is nothing more than the *resultant* of different and contending feelings or "motives," or the *excess* of *some particular motive*, which remains *without an opponent*, in conjunction with the intelligence which guides it. As already observed, however, in cases in which the feeling or impulse to action, as in passion, acquires so much intensity, as to leave no time or opportunity for deliberation, the action at the moment is not a volition at all.

A great deal of confusion of thought and ambiguity of expression have been displayed by different writers, with regard to the relation between "the will" and *desire*. Although I shall have occasion to recur to this subject at a future period, it must now be partially considered.

"Desire and will," says Reid, "agree in this, that both must have an object, of which we must have some conception; and, therefore, both must be accompanied with some degree of understanding. But they differ in several things."* "The object of

---

* *On the Active Powers*, Essay ii. chap. 1. From this passage it is evident desire," he continues, "may be anything which appetite, passion, or affection leads us to pursue. I may desire meat, or drink, or ease from pain; but to say that I *will* meat, or *will* drink, or *will* ease from pain, is not English. There is, therefore, a distinction in common language between *desire* and *will.* And the distinction is, that what we will must be an action, and our own action; what we desire may not be our own action; it may be no action at all."* The distinction here drawn is perfectly clear and correct. But let us examine all the relations between the facts thus distinguished, and see what they involve. When it is said that I desire meat, or drink, or ease from pain, the meaning is, that I have a *craving* for meat, or drink, or ease from pain. Now this same craving or desire, by *reflection through the intellect,* will be *directed toward the means* of obtaining its *primary* object, and, through this, toward *the act* by which it is to be secured; so that *now* the *object*—the *secondary* object—of the desire is, "to act," for the sake of its *primary* object, viz., *the meat, drink,* or *ease from pain;* and if the judgment of the understanding coincide with the *desire to act,* that is, if it excites no other feeling of superior influence, the *act* itself *will* certainly *follow* the desire, as a true *volition,* or act of "the will."

"With regard to our own actions," continues Reid, "we may desire what we do not will, and will what we do not desire; nay, what we have a great aversion to. A man athirst has a strong desire to drink, but, *for some particular reason,* he determines not to gratify his desire." Now of what does his determination consist on this occasion? It is a complex process consisting, 1 of a *decision of the judgment,* that more of *evil* than of *good* of some kind or other, would attend the *indulgence* of the *desire to drink;* which constitutes his "particular reason;" and 2 of a consequent *desire to check* the act of drinking, as a *cause* of the predominant evil. "A man," says Reid, "for health, may take a nauseous draught, for which he has no desire, but a great aversion. Desire, therefore, even when its object is some action of our own, is only an incitement to will, but is not volition."† Now, what are the steps of the process in this act of volition? Simply these: from a *love and desire* of health, with the conviction or hope, that the draught will be the *means* of *restoring* it, he naturally *desires to take* the draught; and this

---

that Reid had a very clear notion of the composite nature of *desire,* as distinguished from a merely *irrational* impulse or instinct, without any conscious end. And Dr. Thomas Brown, in speaking of the same feeling, observes, "It is a feeling which is, of course, in some degree complex, as implying always, together with the vivid feeling that arises on the prospect of good, the conception of the object which seems desirable."—*Philosophy of the Human Mind,* Lecture 65.

* Ibid. † *Loc. Citat.*

desire being stronger than his *aversion* to the *nauseous taste* of the medicine, will prevail, and necessarily result in volition, unless it be checked by some more *influential* desire or feeling excited from without or suggested by reflexion.

Although, therefore, *desire* is not the "will;" although we have many desires that have no immediate reference to *action;* and although we have many desires *to act,* which *do not result in action;* yet, because they are *checked* in *their tendency* by some other desire or feeling which obtains *an ascendancy* either by its *intensity* or *habitual indulgence,* it follows that every *direct* volition, or *express* act of "will," is the *immediate result of a desire,* with the view of procuring some good or avoiding some evil, either for ourselves or others. In fact, the train of thought or reflexion engaged in the process of *deliberation* which renders an action so peculiarly voluntary, is itself *a volition,* and a volition *only because* it is the *immediate result of a desire to think* in that particular way, in consequence of some emotion or feeling, on account of which the deliberation is desired. For without some feeling, emotion, or motive, and the *desire* arising out of it, our thoughts would succeed each other at random, and without any object or end in view. Thus with regard to the influence of our desires over our trains of thought, it is observed by Brown that they "are always followed by the suggestion of images accordant with them. Our mere intention of describing a beautiful landscape, for example, which is but a desire like any other of our desires, is followed by the images of rural beauty, that rise, in succession, to our choice." "The images, in all their variety, arise according to the same simple laws of suggestion. They arise variously, only because a single wish of the mind is varied." "In the whole train of our thought, our conceptions, and the attendant emotions which they induce, still correspond to our prevalent wishes."*

It is not, however, always by its *intensity* as an emotional feeling that one desire acquires an ascendancy over another in volition. For one that is comparatively feeble and calm, in regard to emotion, may control another of much *greater* intensity by the *force of habit* which frequent indulgence *in action* has substituted for that of *feeling.* We know that in learning to execute any voluntary action or series of actions, the difficulty of performing them decreases in the direct ratio of their habitual repetition; and that, therefore, the intensity of the desire and effort required to perform them decreases in the same proportion, until, at length, the actions appear to result from the operation of *thought alone,* without the intervention of a desire.

---

* See Note F, p. 23.

To be *voluntary*, however, they must of necessity be *desired*, or they would be without a conscious purpose or design, and therefore *in*voluntary. This power of controlling a stronger feeling or desire, by one of much less intensity, through the *force of habit* which it engenders, as a substitute for its own, in association with some *guiding thought*, has, I think, tended more than anything else to favour the belief in a *special faculty* of " Will" capable of *controlling* the desires, and entirely independent of them; whereas this very *power of control*, or force of habit, is the accumulated effect of some special emotion and desire, which have *decreased* in intensity just in proportion as the controlling force of habit has been acquired. Hence the importance of being taught from childhood to *love* and *hate*, and *habitually act*, in conformity with the principles of *right* and *wrong*. This, as the foundation of all moral discipline and education, was well understood and strongly inculcated by many of the heathen philosophers. The ethical virtues, says Aristotle, are the effects of habit, as the term ἔθος implies.* Hence he calls virtues laudable habits,† and actions the mistresses of the qualities of habits;‡ and as the whole business of ethics and politics is conversant with pleasures and pains, it is of the greatest importance, as Plato observes, to be so educated from childhood as to be delighted and pained with those things from which *we ought* to feel pleasure and pain, for this is right education.§ For life is a certain energy, and every one energizes in consequence of those things which he particularly loves or hates. And again, as he argues with his usual logical precision, " What affirmation and negation are in the reasoning or judgment, the same are pursuit and avoidance in the craving or appetite (ὄρεξίς). Hence, since ethical virtue is a deliberate habit, and deliberate choice is an appetite in conjunction with consultation, it is necessary that the reason or judgment be true, and the appetite right, if the deliberate choice is good, and that what the one asserts the other should pursue."|| It is important, therefore, not only that the intellect be cultivated and enlightened in accordance with right reason, but that the appetites and affections be at the same time directed in conformity with it, and so strongly associated in action, that the

* *Ethic. Nicom.*, lib. ii. cap. i.

† Ibid. lib. i. cap. vii. ‡ Ibid. lib. ii. cap. ii.

§ *Ethic. Nicom.*, lib. ii. cap. iii. In another place it has been finely observed by Plato, that pleasure and pain are two fountains set flowing by Nature, from which whatever is drawn at the proper place and time and in proper quantity, bestows happiness, and conversely, whether on a private citizen, a state or an animal. Δύο γάρ αὗται πηγαὶ μεθεῖνται φύσει ῥεῖν, κ.τ.λ. De Legg. lib. i.

|| Ibid., lib. vi. cap. ii.

one shall always pursue whatever be indicated by the other.* Xenophon attributes the remarkable virtues and qualities of Cyrus to the education which he received under the institutions and laws of the Persians. "These laws seem to begin with a provident care of the common good; not where those of most other governments begin; for most other governments, giving to all a liberty of educating their children as they please, do then enjoin their people not to steal, not to plunder, &c.; and if they transgress, they impose punishments on them: but the Persian laws, taking things higher, are careful from the beginning to provide that their citizens shall not be such as to be capable of meddling with any action that is base and vile. The boys who frequent the public places of instruction, pass their time in *learning justice;* and tell you that they go for that purpose, as those with us who go to learn letters, tell you that they go for that purpose. Their rulers, for the most part of the day, continue dispensing justice among them, for as amongst the men, so the boys have against each other their accusations of theft, robbery, violence, deceit, and calumny, and other such things as naturally occur; and when they find any one acting unjustly, in any of these ways, they punish them; they punish likewise such as they find guilty of false accusation; and whomsoever they find able to return a benefit, and refusing to do so, they punish severely; for they are of opinion that the ungrateful are careless and neglectful both of the gods, of their parents, of their country, and of their friends."† In a similar way they were taught prudence, modesty, temperance in eating and drinking, obedience to their rulers, with all the accomplishments and arts that could render them good and useful citizens.

---

### Note A.

In a work recently published on "Mental Philosophy," Mr. Morell has modified his earlier opinions on the nature of sensation and its relation to intelligence. "The theory we have propounded," he says, "enables us to draw a clear line of separation between sensation, properly so called, and all the *subsequent mental phenomena* which attach themselves to it." p. 77. "Sensation, taken alone, is not *knowledge;* is not even

---

* "Travaillons donc," says Pascal, "à bien penser: voilà le principe de la morale."—*Pensées,* Art. iv. This is true only when the affections habitually conform to the dictates of right reason; and constitutes therefore only one element in the principles of morals.

† Xenophon. *De Cyri Institutione,* lib. i. p. 10. Hutchinson's Edition. The above extract is from a translation by the Hon. Maurice Ashley Cooper, which is almost literal. P. 4.

*experience*—all that any number of sensations (independently of some *subsequent mental activity*) *could* indicate would be a succession of isolated mental feelings, having no connexion with each other, and leading consequently to *no kind of intelligence* or knowledge." p. 81. And again, "The physical process terminating in a state of *passive feeling* is all that we include under the term *sensation;* the *mental activity* which commences the moment a second state of consciousness is experienced is the first step in the development of perception." p. 83. Compare this passage with my own remarks on Sir Wm. Hamilton's opinion, at page 578 of my former article. Both that article and the present were *written* before the *publication* of Mr. Morell's "Mental Philosophy," and the former one was *printed* before I had an opportunity of seeing that work.

Note B.

When the craving of appetite becomes so urgent that it hurries on to action, without affording the understanding an opportunity of exercising its independent functions in exciting some other dynamic feeling or impulse, by which its tendency can be opposed, the action may be called *involuntary;* but it is not, therefore, the less true, that a *voluntary* action is the immediate *effect of a desire to perform it.* The term "desire" is frequently understood to signify the merely subjective and irrational feeling of appetite in general, or the *mere feeling of attraction* towards its object. Thus *hunger* is called a "desire for food" in general; and the mere *feeling of attraction* of the same appetite towards a present object of food, is called a "desire" for that object. But this is a very different thing from *a craving or desire to perform a definite action,* under the guidance of intelligence, with the conscious design of gratifying the *appetite* or "desire," and with the privilege of considering its consequences.

Aristotle, also, although not always quite consistently, has drawn some good distinctions between the operations of *rational* and *irrational* "desire." For the merely subjective feeling of appetite in general, he employed the word ἐπιθυμία; while by the term ὄρεξις he meant the *conative energy,* the *striving* or *stretching after* an *object* of the ἐπιθυμία. This ὄρεξις, or stretching after, is described by Aristotle as either *rational* or *irrational.* The first corresponds exactly to the craving of "desire" directed by intelligence to a conscious end, and is called βούλησις, which is always employed to signify the "Will;" εστι δὲ ἡ μὲν βούλησις, μετὰ λόγου ὄρεξις αγαθοῦ.*

* *Rhetoric,* lib. i. cap. x.

The term βούλησις also signifies a *wish.** Hence Aristotle sometimes uses βούλησις and ὄρεξις in the same sense. "When craving (ὄρεξις)" he says "moves in accordance with *reason*, it moves also in accordance with the '*will;*' but *craving* may move *without reason ;* for *appetite* (ἐπιθυμία) is a certain *craving.*† The *irrational* ὄρεξεις, or cravings, were rage and appetite (ἄλογοι δ' ὀρέξεις, ὀργὴ καὶ ἐπιθυμία).‡ The schoolmen likewise made a distinction between "Appetitus Sensitivus," and "Appetitus Rationalis." The latter they employed in the same sense as "Will."

NOTE C.

The volitional effect or the *resultant* of the dynamic and contending feelings (referred to at p. 7), considered *simply as forces*, without reference to their *nature*, is similar to the *resultant* of the composition of *physical* forces ; and if the *necessary data* could be obtained, the *reciprocal effects of the organic actions* in the nervous centre through which they are manifested might doubtless be calculated with the greatest precision, and expressed in the same exact mathematical formulæ as are the corresponding effects in *physical* dynamics, or the phenomena of general mechanics, of electricity, and of chemical affinity so far as the necessary data will permit.

The *emotions* consist of peculiar states of feeling, which are generally either *pleasurable or painful*, but in some instances are of a *mixed*, and even of a *neutral*, character. At first they are excited only by reflecting on the special object of each, in relation either to its own circumstances or to ourselves. Thus, while *pity* or *compassion* can be felt only by considering its object as affected by its own unfortunate condition ; the feeling of *jealousy*, *envy*, or *hatred*, can be excited only by reflecting on its object *as affecting ourselves*, or as in some way opposed to our interests or pleasures. In regard, therefore, to their *mode of excitement* by ideas, they differ essentially from physical sensations, but, in the same respect, bear a considerable resemblance to the bodily appetites as well as to certain organic sensations, as of yawning and even of nausea, which latter, however, may be excited mechanically, as well as by ideas. The bodily appetites, therefore, and the other forms of sensation just mentioned, seem to hold a middle place, or form a transition between physical sensations and emotions. After the emotions have become associated with their objects by reflexion, the mere presence or ideas of the same objects will re-excite them without the mediation of the reasoning process on which they were at

* *Ethic. Nicom.* lib. iii. cap. ii. † *De Animâ*, lib. iii. cap. x.

‡ *Rhetoric*, lib. i. cap. x.

first dependent. They are all accompanied, in a greater or less degree, by *in*voluntary excitement of the bodily organs as their natural or instinctive expression; but besides this, they tend, under the guidance of intelligence, to express themselves in a *voluntary* manner, and are then the dynamic elements of true volition. When their intensity or impulse to action is moderate, so that time and opportunity are allowed for reflecting on the consequences of yielding, or for a process of deliberation, the action performed is voluntary, the result of an *explicit desire to perform it;* but the process is nevertheless of a *reflex* character; for the emotion or dynamic feeling is first excited by the presence or idea of its object, and then re-acts under the guidance of intelligence, in the form of *a desire to produce a certain result.* Sometimes, however, the feeling or impulse to action is so great that it engrosses nearly the whole of the consciousness, without leaving any possibility of independent reflection or deliberation, because it excludes from consciousness all intelligence except the small amount required to guide the impulse to its immediate end. Such an impulse or feeling is an uncontrollable *passion*, and the action which it excites is likewise uncontrollable and therefore *in*voluntary.

Note D.

In *learning* to execute a series of voluntary movements we are urged on by a *desire*, or rather by a *series of special desires, to perform them*—that is, by some *impulse* or *dynamic feeling*, guided to its end by intelligence. At first, the whole consciousness is engaged or concentrated in each impulsive feeling in combination with the idea, or in each *special desire.* This concentration constitutes what is called the "effort," and is strong just in proportion to the difficulty experienced in effecting, in the nervous apparatus through which it acts, those organic changes and associations which are required to produce the necessary muscular co-ordination. In proportion, however, as these organic changes are induced, and the transmission of the impulse is *repeated*, the *less* will be the impulse required; until, at length, the whole series of organic changes will succeed each other without the mediation of any *effort*, except a *general desire* to *perform* the *entire process*, and a *special desire* to start the *first* of the series. Meanwhile, the *ideas of the actions* become more and more strongly associated with the *organic changes* in the nervous centre through which they operated *in combination with the dynamic feeling;* so that afterwards they may start the whole train even in the absence of that *feeling;* but the actions will then be involuntary, although not necessarily uncontrollable—involuntary, however, because they are not executed by the wish or desire of the agent—that is, by that *combination* of *active feeling and intelligence* which constitutes his personality. Our own subjective experience informs us, on reflection, that all *sense of effort* arises out of the exertion of the active or dynamic feeling in combination with intelligence; and it is because the ideas themselves are co-ordinated through the same feeling, that both *voluntary thought* and *action* are attended with a sense of effort.

The opinion of the late Mr. James Mill on the nature of volition appears to coincide with that of Hartley, and, to a certain extent, with that of Mr. Bain. He held that all actions were originally the result of sensations; that by repetition the ideas of the sensations and the ideas of the contractions of the muscles become associated so strongly that the *actual contraction* follows upon the mere *idea* of a sensation. "A sudden sensation," he observes, "of pain in the eye makes the eyelid close. When this has been performed a number of times, the idea of the pain in the eye, and the idea of the contraction of the muscles—that is, of the sensations contained in the contraction of the muscles, become associated together so strongly, that the one can never exist without the other. The next step of the process is, that the contraction follows upon the idea, in the same manner as it followed upon the sensation. And as the idea and the sensation," he continues, "are feelings so nearly alike, there is no difficulty in believing that like effects proceed from like causes."* Now it is quite certain, as I have already stated, that we perform voluntarily a vast number of actions that were never originally associated with either physical *pleasure* or *pain*; and certainly, *so far as such a thing is possible,* I can have an *idea* of a painful impression without its being necessarily followed by the contraction which the actual pain produced. I can think of the painful impression of the sun-light which caused contraction of my eyelids, without any repetition of the contraction. But, according to Mr. Mill, "we never have the *ideas* as *antecedent*, without the movement as consequent. This inseparable connexion," he says, "between the ideas and the contractions, which we call the power of the will, is gradually formed. At first, the hand of the child is moved by sensations."† Such movements, however, would be the result of *uncontrollable* associations, and therefore *in*voluntary. If we never have the *ideas* of a movement as antecedent, without the *movement itself* as consequent, then it would not be possible to call up the ideas, as I have now been doing,

* *Analysis of Phenomena of the Human Mind*, p. 266.
† *Op. cit.*, p. 266.

*for the mere purpose of examination and analysis,* without their exciting the corresponding movements. But some of the movements which Mr. Mill adduces as the results of *ideas alone,* are, in reality, the immediate effects of active feeling or emotion. Such is the winking of the eyelids when a person's hand is rapidly moved before the eyes. "The *idea,*" says Mr. Mill, "is that of pain from contact of the hand with the eye." Now, it is not *simply* the *idea* of pain that causes the winking, but an irresistible *feeling* of *fear arising out of* the idea of *impending* pain, or of pain which the hand may possibly or probably inflict. The feeling *of fear* is, as it were, the representative—through the idea—of the feeling of *pain,* and has the same effect.

The error of Mr. Mill consists in his confounding *ideas* with the *feelings or emotions* which they excite. "An *aversion,*" says he, "is the *idea* of pain; they are not two things, but two names for the same thing." Now, *aversion* is not an *idea* at all, but a *feeling* excited by an idea. Again he says, "The idea of pleasure is the idea of something good to have. But what is *desire* other than the *idea* of something good to have?"* It is surprising that a mind so acute and analytical as that of Mr. Mill should fail to perceive the error involved in these statements. The *idea* of a pleasure, or of something good to have, is the *cause* or the *occasion* of the *feeling* of desire, and not the *desire itself.* Is the *idea* of a loaf the same thing as the *feeling of craving to obtain it?* With the most *vivid idea* of food, there may be an entire absence of *desire,* as in loss of appetite. In a similar way Mr. Mill identified the *emotion of grief* with the *ideas* by which it is excited. "What we call grief," he says, "is the *existence of certain trains of ideas.* The ideas exist; the weeping follows."† True, that the *action of weeping follows* the ideas, but between the two states there intervenes an *emotion,* which is the *proximate* cause of the weeping. Yawning and laughter are also explained by Mr. Mill as the *immediate effects of ideas.* In both cases, however, there is an intervening sensation, which is the *proximate* cause of the action, *whether or not it be excited by an idea.* Yawning may occur as the immediate result of an idiopathic or spontaneous feeling—at least, of a feeling or inclination which is not excited *either through the mind or the senses,* but which, nevertheless, is apparently of a reflex character—that is, the effects of a *re*-action of the sensorial centre, when excited by some impression arising from particular states of the organism,

---

as fatigue, debility, &c. But the same spontaneous and *in*voluntary act of yawning may be rendered *voluntary* simply by the *assent* or *sanction* of the *understanding*. Suppose, for instance, that in the company of strangers, I feel a strong involuntary impulse or inclination to yawn. *A moment's reflection* on my position will excite *a counter-feeling of propriety*, or an *inclination* which, if neglected, would result in *a feeling of shame*, and which, therefore, *opposes* my *inclination to yawn.* Here, then, is *a volitional check* on the *in*voluntary impulse, in the form of *a sense of propriety and dread of shame* excited by reflection on my position. The *restrained* inclination to yawn, however, is a *painful* sensation, arising, probably, from the retention of nerve-force in the sensorial centre; and if this painful inclination should happen to continue, it would itself excite a desire to adopt the means of removing it—as, for instance, by leaving the room—and would, therefore, become the *active* or *dynamic* element of a *voluntary* impulse, with the express design of escaping from the company of strangers, as the *occasion* of the *feeling of propriety*, which, by *opposing* the inclination to yawn, was the *cause* of the *painful restraint.* The restraining influence, therefore, or dread of shame, being no longer *felt*, the act of yawning would of course ensue upon the *originally instinctive inclination;* and, like an action resulting from physical sensation, under similar circumstances, would be rendered *voluntary simply* by the *assent* of the *understanding*—by removal of the check or *opposing feeling* excited by the reflection—or, as would be said in ordinary language, by permission of the "Will."* In such an act of yawning, however, there is no exertion of *effort;* for since the feeling which excites it is naturally associated with the necessary muscular co-ordination, and therefore encounters no obstacle, there is no need of any co-operation of the intellect to guide it. But the sense of effort *arises* from this *co-operation* and *concentration* of *feeling* and *intellect,* in the form of a *special desire to act;* for the combination of feeling and intellect can alone constitute the consciousness of "ourselves" as personal agents, and without this consciousness there can be no sense of *personal effort.* When the sensation of yawning, however, as well as the appetites and emotions, act through the intellect as a volitional feeling of *desire,* they appear to transmit their influence to the muscles through a different channel from that by which they operate instinctively. The impulse of yawning acts through the respiratory nerves, in association with some other of the spinal.

---

* See p. 2.

NOTE E.

This reciprocal transfer or convertibility of dynamic feeling through the medium of ideas might be called the *mediate correlation of æsthetic force.* It may not, perhaps, be improper to mention incidentally the fact, that at an early age, while going through a course of the physical sciences, and five or six years before the first appearance of Mr. Grove's discovery, the whole theory of the *correlation,* or, as I then called it, the *mutual convertibility, of physical forces,* occurred most distinctly to my mind; and well do I recollect, on seeing the "just published" first edition of Mr. Grove's work exposed in Highley's window, the eagerness with which I hastened to purchase it, and the deep interest with which, as I walked down Fleet-street, I ran over some of the experimental proofs of theoretical conclusions that were forming independently in my own mind. Of the *correlation* of physical forces, however, as well as of the *conservation* of force, which is indeed a necessary corollary from the former, I find that Leibnitz had evidently a clear conception. In the first of the papers which passed between himself and Dr. Samuel Clarke, he makes the following remarks on the way in which the Supreme Being maintains the machinery of the universe, and conducts its unceasing operations. "Monsieur Newton, et ses sectateurs, ont encore une fort plaisante opinion de l'ouvrage de Dieu. Selon eux, Dieu a besoin de *remonter* de temps en temps sa montre ; autrement elle cesseroit d'agir. Il n'a pas eu assez de veue, pour en faire un mouvement perpetuel. Cette machine de Dieu est même si imparfaite selon eux, qu'il est obligé de la *décrasser* de temps en temps par un concours extraordinaire, et même de la *raccommoder,* comme un horloger son ouvrage; qui sera d'autant plus mauvais maistre, qu'il sera plus souvent obligé d'y retoucher et d'y corriger. Selon mon sentiment, *la* même *force et vigueur y subsiste toujours, et passe seulement de matiere en matiere, suivant les lois de la nature, et le bel ordre* préétabli."*

NOTE F.

In a foot-note to Chap. I., Essay ii., of Reid on the Active Powers, Sir Wm. Hamilton asserts that *Will* was considered as a mere modification of *Desire* "by Dr. Thomas Brown, in whose scheme there was thus virtually abolished all rational freedom, all responsible agency, all moral distinction." Dr. Brown, however, held no opinion of the kind. It must be recollected that he employed the word *desire* as a generic term for all our prospective emotions: thus, Wish, Hope, Expecta-

* Extrait d'une lettre écrite au mois de Novembre, 1715.

tion, Avarice, Love of Glory, of Knowledge, of Society, &c., were only *different forms of desire.* In no part of his *Lectures* does he give any distinct definition of *will* or *volition;* but in his observations on the Zoonomia of Dr. Erasmus Darwin, Sir Wm. Hamilton might have found the following remarks: "Admitting [with Darwin] love, ambition, avarice, to be names of particular desires; and hatred, disgust, fear, anxiety, of particular aversions, *they are not, therefore, exertions of volition. Volition is not desire itself, but exertion in consequence of desire.* If the desire and the volition were the same, they would usually be followed by fibrous [muscular] motions, for the same effect must result from the same sensorial change. But love, or hatred, may be felt without any fibrous exertion; and the martyr of disease is not precluded from the desire of exercise by being unable to enjoy it." (P. 59, section ii). I have already shown that volition is the immediate result of a *desire to act* which is *not checked* by some stronger, or, at least, *more influential desire,* arising out of some feeling or emotion that *reacts through intelligence* for the attainment or avoidance of its object; and that what is called the *exertion* of volition, or the *sense of effort,* is the coincidence and approval of the intellect in the felt impulse, and the consequent combination and concentration of both in the desire itself, or upon the desired end. (See Note D.) The only difference, then, between an *in*voluntary and a *voluntary* impulse, is that, in the former, although the intellect *disapproves* of the result, the correlative feeling, motive, or emotion which it excites, is insufficient to resist the impulse; while in the latter, the intellect *approving* of the result, excites *no opposing feeling or motive* whatever, but coincides, or goes along, with the impulse, and, together with it, constitutes our consciousness of *personal effort. In the absence of any other opposing and more influential desire* or feeling, *a desire to act* must necessarily, by its *impulsive* or *dynamic tendency, result in action;* the greater *exertion* or effort is the greater concentration of the intellect, in combination with, and as the immediate result of, the desire itself. Whatever mysterious notions Sir William Hamilton might have entertained respecting "the Will," it is pretty certain that he knew little or nothing of the *real process of volition*—certainly less than was known by Brown, whom he seemed to delight in accusing of *mistakes* and *plagiarisms.* (See especially his *Discussions on Philosophy,* &c., art. ii.) But it is rather surprising that while making these accusations, he did not see the impropriety of appropriating some of Brown's thoughts without acknowledging the source whence he derived them. Thus, in correcting Reid's statement, that memory is "an *immediate* knowledge of the *past,*" he shows only what Brown had already shown, that such a knowledge is impossible, and that our knowledge of the *past* can be only *mediate.* (Compare Brown's *Philos. of Human Mind.* Lect. 41.)

(*To be continued.*)

---

### Art. II.—ON COOLING OF THE BODY AFTER DEATH.

